# MRI-Based Predictors of Hemorrhagic Transformation in Patients With Stroke Treated by Intravenous Thrombolysis

**DOI:** 10.3389/fneur.2019.00897

**Published:** 2019-08-27

**Authors:** Rody El Nawar, Jennifer Yeung, Julien Labreuche, Marie-Laure Chadenat, Duc Long Duong, Maxime De Malherbe, Yves-Sebastien Cordoliani, Bertrand Lapergue, Fernando Pico

**Affiliations:** ^1^Department of Neurology and Stroke Center, Hopital Mignot, Centre Hospitalier de Versailles, Versailles, France; ^2^Gilbert and Rose-Marie Chagoury School of Medicine, Lebanese American University Medical Center, Beirut, Lebanon; ^3^Université de Lille, CHU Lille, EA 2694, Santé Publique: Épidémiologie et Qualité des Soins, Lille, France; ^4^Department of Radiology, Centre Hospitalier de Versailles, Versailles, France; ^5^Department of Radiology, Hopital Privé de Parly 2, Versailles, France; ^6^Department of Neurology and Stroke Center, Hopital Foch, Suresnes, France; ^7^Université Versailles Saint-Quentin en Yvelines et Paris Saclay, Versailles, France; ^8^INSERM U1148 LVTS (Laboratory for Vascular Translational Science), Team 5 (Research into “Atherothrombotic Disease in Heart and Brain”), Hôpital Bichat, Paris, France

**Keywords:** hemorrhage, stroke, ischemic, intravenous thrombolysis, magnetic resonance imaging, diffusion-weighted imaging

## Abstract

**Background:** Clinical and biological risk factors for hemorrhagic transformation (HT) after intravenous thrombolysis (IT) have been well-established in several registries. The added value of magnetic resonance imaging (MRI) variables has been studied in small samples, and is controversial. We aimed to assess the added value of MRI variables in HT, beyond that of clinical and biological factors.

**Methods:** We enrolled 474 consecutive patients with brain infarction treated by IT alone at our primary stroke center between January 2011 and August 2017. Baseline demographic, clinical, biological, and imaging characteristics were collected. MRI variables were: brain infarction volume in cm^3^; parenchymal fluid attenuated inversion recovery (FLAIR) hyperintensity; FLAIR hyperintense vessel signs; number of microbleeds; subcortical white matter hyperintensity; and thrombus length.

**Results:** Overall, 301 patients were included out of 474 (64%). The main causes of exclusion were combined thrombectomy (*n* = 98) and no MRI before IT (*n* = 44). In the bivariate analysis, HT was significantly associated with the presence of more FLAIR hyperintense vessel signs, thrombus length (>8 mm), and larger brain infarction volume (diffusion-weighted imaging (DWI) and apparent diffusion coefficient (ADC) < 500 × 10^−6^ mm^2^/s). In the multivariable analysis, only brain infarction volume was significantly associated with HT. The discrimination value of the multivariable model, including both the DWI volume and the clinical model (area under the receiver operating characteristic curve, 0.80; 95% confidence interval 0.74–0.86), was improved significantly compared with the model based only on clinical variables (*P* = 0.012).

**Conclusions:** Brain infarction volume on DWI was the only MRI variable that added value to clinico biological variables for predicting HT after IT.

## Introduction

The most feared complication after intravenous thrombolysis (IVT) with tissue-type plasminogen activator is cerebral hemorrhagic transformation (HT) (1–3). Many clinical, biological, and computed tomography (CT) factors have been associated with symptomatic HT in several registries, such as SITS-MOST (Safe Implementation of Thrombolysis in Stroke: Monitoring Study) (4). A variety of scores and nomograms have been developed to predict the risk of HT after IVT (5–7). Most previous studies were based on radiological variables assessed by CT and CT angiography (8–12). Magnetic resonance imaging (MRI) variables have been studied in small samples, with only one or two variables analyzed, and their added value above clinical and biological variables has not been assessed; therefore, this issue is controversial in the literature. The aim of this study was to assess the added value of MRI variables in HT, taking into account proven clinical and biological factors.

## Methods

### Subjects and Methods

We identified from our prospective IVT registry, patients presenting with an acute ischemic stroke to our primary stroke unit, between January 2011 and June 2017. A total of 474 consecutive patients with brain infarction treated by IVT alone were enrolled. Since 2015, patients with a proximal artery occlusion have been transferred directly for thrombectomy; these patients were excluded from our study, to avoid heterogeneous treatment.

All the included patients received only IVT according to the European license (alteplase 0.9 mg/kg, given intravenously; maximum dose, 90 mg) after a confirmed brain MRI. After administration of the initial bolus dose (10% of the total dose), the remainder was infused over 1 h. HT was assessed within 24 h after the IVT, with a CT scan or brain MRI, using the ECASS II classification (1).

For each patient, we collected socio demographic data, previous medical conditions, vascular risk factors, disability before stroke (assessed by a modified Rankin disability scale), medication, laboratory data (leucocyte, neutrophil, and platelet counts, hematocrit, low-density lipoprotein cholesterol, glycemia), vital signs, National Institutes of Health Stroke Scale (NIHSS) score on admission, cause of the acute ischemic stroke based on the TOAST criteria (13), and time from onset of symptoms to bolus of IVT.

The brain MRIs were performed with a1.5T MRI machine (GE Healthcare, Chicago, IL, USA), according to the acute stroke protocol. This protocol included diffusion-weighted imaging (DWI) at B1000 and apparent diffusion coefficient (ADC) sequences, a fluid attenuated inversion recovery (FLAIR) sequence, a T2^*^ sequence, and time-of-flight angiography. Each sequence was composed of 20 interleaved slices of 5 mm slice thickness, with an interslice space of 6.5 mm, except for 97 sets of time-of-flight images of 1.4 mm slice thickness and interspaced by 0.7 mm. The field of view was 128 × 128 cm for DWI and ADC, 320 × 224 cm for FLAIR, 384 × 224 cm for T2^*^, and 288 × 224 cm for time-of-flight angiography. The images were analyzed by one experimented neurologist (R.E.N.), who was blinded to the clinical data. Two experimented neuroradiologists (Y.-S.C. and M.D.M.) trained the neurologist (R.E.N.) and validated the process for collecting MRI data.

The following MRI brain variables were analyzed: stroke volume on DWI at B1000 and ADC < 500 × 10^−6^ mm^2^/sequences in cm^3^, the presence or absence of FLAIR hyperintensity, the presence or absence of FLAIR hyperintense vessel sign (FHVS) [considered positive when focal or tubular hyperintensities were present in the subarachnoid space, and classified from 0 to 10 depending on the number of positive slices (14)], microbleeds, subcortical white matter hyperintensity (leukopathy) rated according to the Fazekas scale (15), measurement of the thrombus length when present on the T2^*^ sequence, and localization of the arterial occlusion and the arterial territory involved in the stroke. Stroke volumes were measured manually on the DWI sequences, with ADW Server 2.0 software (Vosselaar, Belgium), on different slices, and then the volume was calculated automatically in cm^3^. Likewise, the stroke volumes on the ADC < 500 × 10^6^ mm^2^/s sequences were calculated in the same way, with the ADC technique. HT was classified according to the ECASS II classification (1). For the purpose of this study, all types of HT were considered and clustered into the variable named HT.

### Ethics

In accordance with French legislation, this study did not need approval by an ethics committee or written informed consent from patients because it only encompassed the analysis of anonymized data that had been collected prospectively as part of routine clinical care. Moreover, in both the stroke center and the medical record sent to the patients and their general practitioners, the patients were informed about their right to refuse the use of the anonymized data for research purposes.

### Statistical Analysis

Categorical variables are expressed as frequencies and percentages. Continuous variables are expressed as means ± standard deviations or medians [interquartile ranges (IQRs)] for non-normal distribution. Normality of distributions was assessed graphically, and by using the Shapiro-Wilk test. We first assessed the association of baseline demographic, clinical, and biological characteristics with HT after IVT in bivariate analyses, using the χ^2^ test or Fisher's exact test for categorical characteristics, and Student's *t* testor the Mann-Whitney *U* test for continuous characteristics, as appropriate. Characteristics associated with HT after IVT in bivariate analyses (*P* < 0.10) were included in a multivariable logistic regression model. Before developing the multivariable prognostic model (namely the clinical model), continuous predictors were categorized using quartiles to examine the log-linearity assumption, and collinearity between candidate predictors was examined by calculating the variance inflation factors. The performance of the multivariable clinical model was examined in terms of calibration using the Hosmer-Leme show goodness-of-fit test, and discrimination using the area under the receiver operating characteristic curve (AUC). Secondly, we assessed the prognostic values of MRI lesions (after log-transformation of lesion volumes) on HT after IVT in logistic regression models, before and after adjustment for the clinical model; comparisons of AUCs were performed using the DeLong approach. Spearman's rank correlation coefficients between significant MRI lesions were calculated. Statistical testing was done at the two-tailed α level of 0.05. Data were analyzed using the SAS software package, release 9.4 (SAS Institute, Cary, NC).

## Results

**Figure 1:**
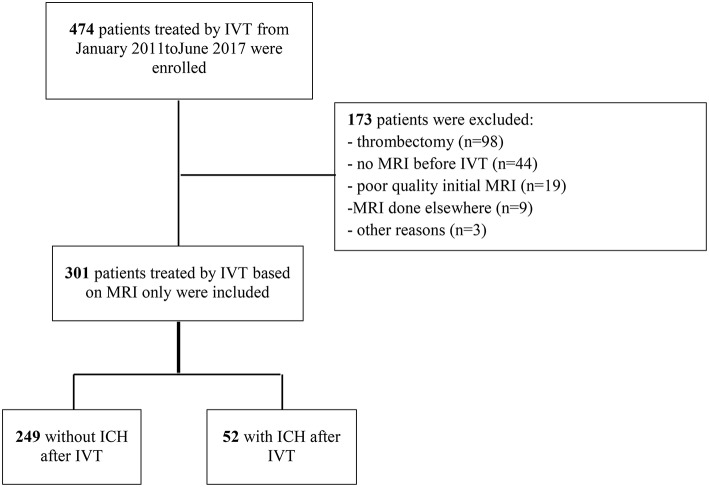
Study flow chart. ICH, intracranial hemorrhage; IVT, intravenous thrombolysis; MRI, magnetic resonance imaging.

Between January 2011 and August 2017, 474 patients were treated consecutively by IVT in our center; of these, 301 patients with IVT based on MRI findings were eligible to be included in the present study (Figure 1). Reasons for exclusions were: absence of brain MRI before IVT (*n* = 44); thrombectomy (*n* = 98); poor quality or absence of control brain MRI or CT scan (*n* = 19); MRI done at a different institution (*n* = 9); and other reasons (*n* = 3). The main baseline characteristics of patients included and not included are reported in the online-only Data Supplement (Table S1). The strongest difference between included and excluded patients was observed for baseline stroke severity: less severe clinical severity was detected in included patients, with a median admission NIHSS score of 9 [IQR, 6–15] compared with 16 [IQR, 11–21] in excluded patients. HT within 24 h occurred more frequently in excluded patients (44.2%; 95% confidence interval [CI],36.7–51.6%) than in included patients (17.3%; 95%CI, 13.0–21.6%; *P* < 0.001). A similar difference was observed with symptomatic HT: 23.2% (95%CI, 15.8–30.6%) in excluded patients compared with 6.4% (95% CI, 3.4–9.4%) in included patients (*P* < 0.001).

### Intracranial Hemorrhage, and Baseline Clinical and Biological Characteristics

Table 1 shows the main baseline clinical and biological characteristics of patients treated by MRI-based IVT, according to the occurrence or not of HT within 24 h. In the bivariate analysis, HT after IVT was significantly associated with older age, no current smoking, higher admission NIHSS score, use of antithrombotic medication before stroke event, and atrial fibrillation history. In multivariate regression analysis including age, smoking, admission NIHSS score, use of antithrombotic medication, and atrial fibrillation, only the prognosis effect of admission NIHSS score remained significant (OR per 5-point increase, 1.47; 95%CI, 1.16–1.86; *P* < 0.001). The discrimination of this multivariable prognostic model was 0.72 (95%CI, 0.64–0.80), with a good calibration, as indicated by the Hosmer-Leme show goodness-of-fit test (*P* = 0.71).

**Table 1:** Baseline characteristics according to occurrence of ICH after IVT.

	**No ICH (*n* = 249)**	**ICH (*n* = 52)**	***P* Values**
Age, median [IQR], y	74 [62–83]	80 [70–87]	0.011
Men, *n*/total *n* (%)	130/249 (52.2)	26/52 (50.0)	0.77
**Medical history, *n*/total *n* (%)**			
Hypertension	161/249 (64.7)	38/52 (73.1)	0.24
Diabetes	40/249 (16.1)	10/52 (19.2)	0.58
Hypercholesterolemia	96/249 (38.6)	23/52 (44.2)	0.45
Current smoking	61/249 (24.5)	6/52 (11.5)	0.041
Coronary artery disease	39/249 (15.7)	10/52 (19.2)	0.53
Previous stroke	50/249 (20.1)	8/52 (15.4)	0.43
Atrial fibrillation	43/249 (17.3)	19/52 (36.5)	0.002
**Current stroke event**			
NIHSS score, median [IQR]	8 [5–14]	14 [7–20]	<0.0001
Prestroke mRS ≥ 1, *n*/total *n* (%)	38/249 (15.3)	9/51 (17.7)	0.67
Statin users, *n*/total *n* (%)	78/248 (31.5)	18/52 (34.6)	0.66
Antithrombotic medications, *n*/total	90/249 (36.1)	28/52 (53.9)	0.017
*n* (%)			
Aspirin	71/249 (28.5)	21/52 (40.4)	0.091
Clopidogrel	16/249 (6.4)	4/52 (7.7)	0.76
Anticoagulants	10/249 (4.0)	4/52 (7.7)	0.27
SBP^*^, mean ± SD, mmHg	154 ± 26	157 ± 29	0.55
DBP^*^, mean ± SD, mmHg	82 ± 14	83 ± 19	0.81
**Biological data**			
LDL-C^†^, median [IQR], g/L	1.10 [0.89–1.40]	1.10 [0.85–1.22]	0.49
Blood glucose^‡^, median [IQR], g/L	1.20 [1.00–1.42]	1.24 [1.13–1.56]	0.10
White blood cells^§^, mean±SD, g/L	8.5 (2.7)	8.1 (2.5)	0.39
Platelet count^||^, median [IQR], g/L	230 [188–268]	217 [179–276]	0.48
Neutrophils^||^, mean ± SD, g/L	5.8 (2.7)	5.6 (2.4)	0.67
Hematocrit^||^, mean ± SD, %	41.9 (5.3)	41.5 (4.6)	0.62
Onset to IVT, median [IQR], min	155 [130–195]	160 [150–210]	0.17
Wake-up stroke, *n*/total *n*, %	49/249 (19.7)	12/52 (23.1)	0.58

*DBP, diastolic blood pressure; ICH, intracranial hemorrhage; IQR, interquartile range; IVT, intravenous thrombolysis; LDL-C, low-density lipoprotein cholesterol; NIHSS, National Institutes of Health Stroke Scale; mRS, modified Rankin scale; SBP, systolic blood pressure; SD, standard deviation*.

* *n = 15 missing values*.

† *n = 12 missing values*.

‡ *n = 4 missing values*.

§ *n = 6 missing values*.

|| *n = 7 missing values*.

### Intracranial Hemorrhage and MRI Variables

**Figure 2:**
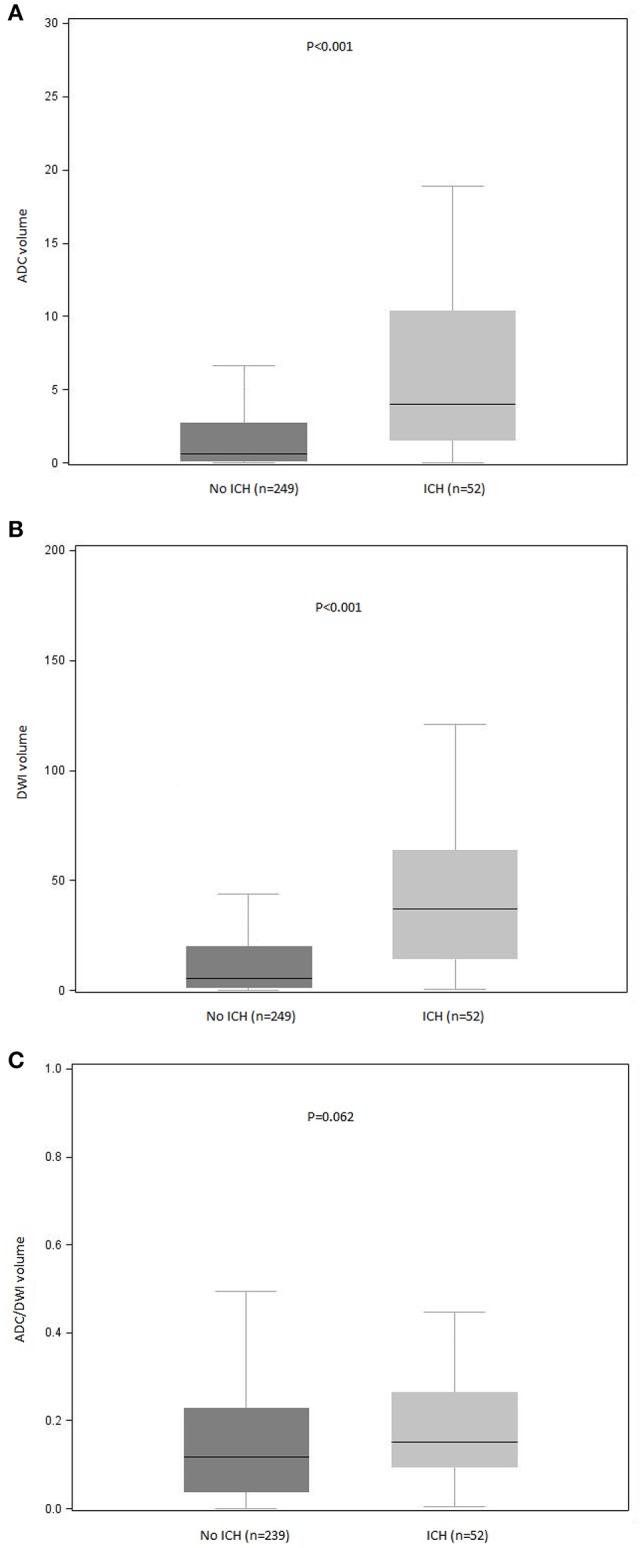
Distribution of **(A)** ADC, **(B)** DWI, and **(C)** ADC/DWI volumes according to occurrence of ICH after intravenous thrombolysis. ADC, apparent diffusion coefficient; DWI, diffusion-weighted imaging; ICH, intracranial hemorrhage. *P* values for between-group comparisons are reported (Mann-Whitney *U* test). Bars indicate the median with interquartile range values. Boxes show the 25th, 50th, and 75th percentiles, and whiskers show the 5th and 95th percentiles.

**Figure 3:**
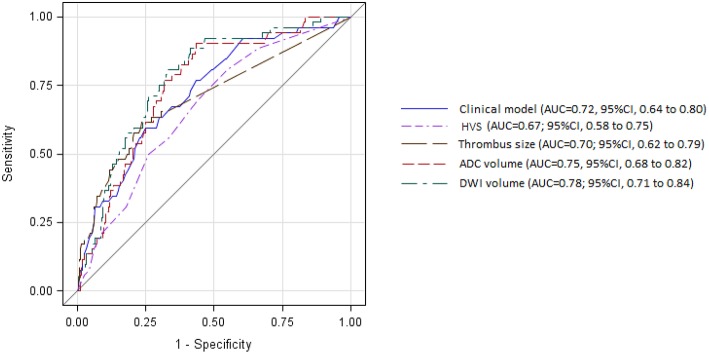
Receiver operating characteristic curves for prediction of intracerebral hemorrhage after intravenous thrombolysis by clinical model and magnetic resonance imaging variables. ADC, apparent diffusion coefficient; AUC, area under the curve; CI, confidence interval; DWI, diffusion-weighted imaging; HVS, hyperintense vessel sign.

In the bivariate analysis, HT after IVT was significantly associated with more FLAIR hyperintense vessel sign, larger thrombus size, and larger volumes on brain MRI DWI and ADC sequences (Table 2 and Figure 2). A gradual increase in the rate of HT after IVT was found with increasing severity of brain MRI variables. The strongest discrimination was found with DWI (AUC, 0.78; 95% CI, 0.71–0.84) and ADC (AUC, 0.75; 95% CI, 0.68–0.82; Figure 3); these two brain MRI variables were strongly correlated (*r* = 0.84). ADC and DWI volumes had a low positive correlation with FHVS and thrombus size (*r* < 0.40). A low positive correlation was also found between FHVS and thrombus size (*r* = 0.42).

**Table 2:** Association between MRI lesions and ICH after IVT.

	**No ICH (*n* = 249), *n*/total *n* (%)**	**ICH (*n* = 52), *n*/total *n* (%)**	**Unadjusted OR (95% CI)**	***P* Values**	Adjusted OR (95% CI)^*^	***P* Values**
PFH	42/249 (16.9)	12/52 (23.1)	1.48 (0.71–3.06)	0.29	1.78 (0.71to 3.89)	0.15
HVS			1.21 (1.09–1.34)^†^	<0.001	1.15(1.03–1.29)^†^	0.005
0	83/247 (33.6)	6/52 (11.5)	1.00 (ref.)	–	1.00 (ref.)	–
1–2	56/247 (22.7)	10/52 (19.2)	2.47 (0.85–7.19)	0.097	2.03 (0.67–6.10)	0.21
3–5	64/247 (25.9)	20/52 (38.5)	4.32 (1.64–11.39)	0.003	3.57(1.30–9.78)	0.013
6–10	44/247 (17.8)	16/52 (30.8)	5.03 (1.83–13.77)	0.002	3.14(1.08–9.07)	0.035
≥1 CMB	64/248 (25.8)	11/52 (21.2)	0.77 (0.37–1.60)	0.48	0.71 (0.33–1.54)	0.40
Thrombus			1.19 (1.11–1.27)^†^	<0.001	1.16 (1.08–1.25)^†^	<0.001
No thrombus	166/248 (66.9)	18/52 (34.6)	1.00 (ref.)	–	1.00 (ref.)	–
≤8 mm	63/248 (25.4)	16/52 (30.8)	2.34 (1.12–4.88)	0.023	1.94(0.90–4.17)	0.089
>8 mm	19/248 (7.7)	18/52 (34.6)	8.74 (3.89–19.59)	<0.001	6.87(2.86–16.48)	<0.001
Leukopathy			1.26 (0.96–1.66)^†^	0.092	1.03(0.73–1.45)^†^	0.86
0	65/249 (26.1)	9/51 (17.7)	1.00 (ref.)	–	1.00 (ref.)	–
1	87/249 (34.9)	18/51 (35.3)	1.49 (0.63–3.54)	0.36	1.03(0.38–2.79)	0.95
2	40/249 (16.1)	6/51 (11.8)	1.08 (0.35–3.28)	0.89	0.53(0.14–1.96)	0.34
3	57/249 (22.9)	18/51 (35.3)	2.28 (0.95–5.75)	0.065	1.13(0.374–3.40)	0.83
**Infarct side**						
Right	102/246 (41.5)	19/51 (37.2)	1.00 (ref.)	–	1.00 (ref.)	–
Left	127/246 (51.6)	31/51 (60.8)	1.31 (0.70–2.46)	0.40	1.06 (0.54–2.06)	0.87
Bilateral	17/246 (6.9)	1/51 (2.0)	0.32 (0.04–2.52)	0.28	0.22 (0.02–1.80)	0.16
**Lesion volume**						
ADC <500 × 10^−6^ mm^2^/s			1.92 (1.48–2.48)^†^	<0.001	2.05(1.46–2.88)^†^	<0.001
<0.13 cm^3^	73/249 (29.3)	3/52 (5.8)	1.00 (ref.)	–	1.00 (ref.)	–
0.13–0.81 cm^3^	70/249 (28.1)	4/52 (7.7)	1.39 (0.30–6.44)	0.67	1.38 (0.29–6.50)	0.69
0.82–4.08 cm^3^	56/249 (22.5)	20/52 (38.5)	8.69 (2.45–30.71)	<0.001	7.94(2.11–29.80)	0.002
>4.08 cm^3^	50/249 (20.1)	25/52 (48.1)	12.17 (3.48–42.48)	<0.001	13.16(3.36–51.46)	<0.001
DWI			2.09 (1.62–2.69)^†^	<0.001	2.25(1.62–3.12)^†^	<0.001
<2.1 cm^3^	73/249 (29.3)	2/52 (3.9)	1.00 (ref.)	–	1.00 (ref.)	–
2.1–7.6 cm^3^	71/249 (71)	4/52 (7.7)	2.06 (0.36–11.59)	0.41	2.11(0.36–12.14)	0.40
7.7–28.7 cm^3^	59/249 (23.7)	17/52 (32.7)	10.52 (2.33–47.36)	0.002	10.54(2.24–49.56)	0.003
>28.7 cm^3^	46/249 (18.5)	29/52 (55.8)	23.01 (5.24–101.05)	<0.001	24.13(4.86–119.63)	<0.001
ADC <500/DWI			2.18 (0.31–15.53)^†^	0.44	1.64 (0.21–13.00)^†^	0.64
<0.047	65/239 (27.2)	7/52 (13.5)	1.00	–	1.00	–
0.048–0.124	59/239 (24.7)	14/52 (26.9)	2.20 (0.83–5.83)	0.12	1.67 (0.59–4.67)	0.33
0.125–0.236	58/239 (24.3)	15/52 (28.9)	2.40 (0.91–6.30)	0.075	2.46(0.89–6.768)	0.081
>0.236	57/239 (23.9)	16/52 (30.8)	2.61 (1.00–6.79)	0.050	2.41(0.86–6.69)	0.092

*ADC, apparent diffusion coefficient; CI, confidence interval; CMB, cerebral microbleed; DWI, diffusion-weighted imaging; HVS, hyperintense vessel sign; ICH, intracranial hemorrhage; IVT, intravenous thrombolysis; MRI, magnetic resonance imaging; OR, odds ratio; PFH, parenchymal fluid attenuated inversion recovery(FLAIR) hyperintensity*.

* *Adjusted for age, smoking, admission NIHSS score, use of antithrombotic medication, and atrial fibrillation*.

† *Odds ratio per 1 unit increase associated with MRI variables treated as continuous variables in logistic regression models (after log-transformation for ADC and DWI volumes)*.

Compared with the clinical model, the AUC of each MRI variable did not differ significantly (Figure 3, all *P* > 0.22). In the multivariable logistic regression analyses adjusted for previous multivariable clinical model, the association of HT after IVT with increasing severity of brain MRI variables remained significant (all *p*-value for Hosmer-Leme show goodness-of-fit test > 0.24). The discrimination value of the multivariable model, including both DWI volume and the previous clinical model (AUC, 0.82; 95% CI, 0.5–0.87) was significantly improved compared with the model based only on clinical variables (*P* = 0.003), or on DWI volume (*P* = 0.046).

## Discussion

Our study aimed to investigate the added value conferred by MRI variables in HT after IVT, beyond clinical and biological factors. Between January 2011 and August 2017, we enrolled 474 consecutive patients presenting with an acute ischemic stroke and treated by IVT alone. The first interesting point underlined by the flow chart was that patients with stroke who could have a MRI and were treated by IVT alone had less severe symptoms than those who underwent a CT scan or were treated by IVT and mechanical thrombectomy; therefore, they were less prone to present HT. In our study patient treated by MT or by both MT and IVT have been excluded, because the aim of this study was to assess the added valued of MRI variables in HT in patients treated only by IV thrombolysis. We observed a higher risk of HT in patients treated by MT due to several factors such as higher NIHSS, larger ischemic stroke territory, cardioembolic etiology. Risk factors of HT in patients treated by MT or MT and IVT is a topic of interest but it was not the aim of our study. This point will be investigated in an another study. Secondly, although numerous brain MRI variables have been associated with HT after IVT, only the volume of brain infarction on DWI (B1000 or ADC < 500 × 10^−6^ mm^2^/s) appeared to have added value with respect to the clinical variables. The volume of brain infarction on DWI (B1000 or ADC < 500 × 10^−6^ mm^2^/s) seems to be the most robust prognostic indicator of HT after IVT compared with other radiological variables. In our study we found a gradual increase in the rate of HT after IVT associated with increasing severity of brain MRI variables.

In our study we had 14 patients who presented a symptomatic HT. Therefore, to perform a multivariate analysis we grouped all HT together as the main outcome. As reported in previous studies, both PH and HI are associated with a lower rate of good neurologic outcome (16).

In studies published previously, the added value of MRI variables has been studied in small samples, and is controversial. For example, a retrospective study of 55 patients showed that DWI lesion volumes and ADC values had no strong relationship with HT (17). In contrast, another retrospective study of 27 patients presenting an acute ischemic stroke evaluated with brain MRI ≤ 8 h after symptom onset supported a potential role for DWI in predicting HT after acute ischemic stroke (18). After inducing an experimental stroke in 14 rabbits, Adami et al. associated the initial lower ADC values with an increased risk of secondary HT, and suggested that DWI is of help in predicting both HT occurrence and severity (19).

In addition, our study flow chart showed that patients who did not undergo initial brain MRI had ~3-fold the risk of HT compared with those who underwent a CT brain scan. This can be explained by a higher clinical severity (decreased level of consciousness, agitation, or unstable state) and larger brain infarcts. Several prognostic scores predicting the risk of HT after IVT, such as HAT, DRAGON, Stroke-TPI, SPAN-100, MSS, SEDAN, SITS-ICH, iScore, and ASTRAL, include clinical severity and patient age as major variables. Three of these scores (DRAGON, HAT, and SEDAN) contain radiological variables based on CT as major components (20). None of these scores includes MRI variables as major prognostic variables of HT after IVT. Of note our AUC (i.e., 0.78), was higher to the AUC reported in SEDAN (0.66) and HAT (0.66) but inferior to the AUC of DRAGON score (0.85) (21–23).

In comparison with previous studies, we also found that older age, antithrombotic medication before stroke onset, high NIHSS score on admission, and cardioembolic strokes had a higher risk of symptomatic HT (24–28). Conversely, current smoking seemed to have a protective effect with regard to the risk of HT after IVT (the so-called smoking paradox) (26, 29). These findings argue for the external validity of our results.

Furthermore, our study showed that HT after IVT was significantly associated with the presence of more FHVS, thrombus length (>8 mm), and larger brain infarction volume (B1000 and ADC < 500 × 10^6^ mm^2^/s).Although the volume of the ADC < 500 × 10^6^ mm^2^/s sequence is strongly correlated to HT, we suggest that it is a waste of time in the acute window phase, because it takes about 4 min to calculate this volume, which is strongly correlated with the DWI at B1000 volume.

Interestingly, the patients who had three or more FHVS positive slices on FLAIR sequences were those who had more HT. These data add further support to a previous report by Lee et al. that mentioned the existence of an association between distal FHVS and large middle cerebral infarction, measured on diffusion- and perfusion-weighted MRI images (30). This would support the relationship between FHVS and impairment of the retrograde leptomeningeal collaterals flow that can aggravate the blood supplementation of the ischemic area, and could be considered a predictor of poor outcome after IVT. The number of FHVSs is still controversial as a risk factor for symptomatic HT in the literature, and has only been reported in small studies (14, 30–32).

Our study has several limitations. First, although the register of patients with stroke undergoing IVT was prospective, most of the data collection was performed retrospectively, based on medical reports. Second, DWI and ADC volumes were measured manually, which can lead to time being wasted during the acute window phase after acute ischemic stroke onset. It will be important in future to measure these volumes more accurately and rapidly using automated software. Third, time between symptom onset and imaging was not available for all patients in our register, therefore we couldn't adjust on this parameter.

In conclusion, brain infarction volume on DWI appears to be the only MRI variable with an added predictive value in HT compared with clinico biological variables. Neurologists and practitioners in emergency departments should be aware of the higher risk of symptomatic HT in patients who are unable to undergo MRI brain after an acute ischemic stroke.

## Data Availability

The raw data supporting the conclusions of this manuscript will be made available by the authors, without undue reservation, to any qualified researcher.

## Ethics Statement

In accordance with French legislation, this study did not need approval by an ethics committee or written informed consent from patients because it only encompassed the analysis of anonymized data that had been collected prospectively as part of routine clinical care. Moreover, in both the stroke center and the medical record sent to the patients and their general practitioners, the patients were informed about their right to refuse the use of their anonymized data for research purposes.

## Author Contributions

RE, JY, BL, and FP designed the model of the study. M-LC, DD, MD, and Y-SC provided critical feedback, helped shape the research, and analyzed the data. JL performed the calculations and statistics. RE and FP wrote the manuscript with input from all authors and were in charge of overall direction and planning. RE submitted the manuscript. FP supervised the project.

### Conflict of Interest Statement

The authors declare that the research was conducted in the absence of any commercial or financial relationships that could be construed as a potential conflict of interest.
