# Supplementary material for: MRI-Based Predictors of Hemorrhagic Transformation in Patients With Stroke Treated by Intravenous Thrombolysis
Source: Front Neurol. 2019 Aug 27;10:897. doi: 10.3389/fneur.2019.00897 (PMC6719609; doi:10.3389/fneur.2019.00897)
Supplement: Supplementary file 1 [file Table_1.docx]

**Supplemental Material**

| **Table I. Main Baseline Characteristics and Hemorrhagic Complications in Included and Excluded Patients** | | | |
| --- | --- | --- | --- |
|  | Not included (n=173) | Included (n=301) | *P* Values |
| Age, mean±SD, y | 71.7±14.1 | 71.3±15.9 | 0.99 |
| Men, n (%) | 95 (55.2) | 156 (51.8) | 0.48 |
| Medical history, n (%) | | | |
| Hypertension | 101 (59.1) | 199 (66.1) | 0.13 |
| Diabetes | 25 (14.6) | 50 (16.6) | 0.57 |
| Hypercholesterolemia | 57 (33.3) | 119 (39.5) | 0.18 |
| Current smoking | 44 (27.0) | 60 (19.9) | 0.082 |
| Coronary artery disease | 39 (22.8) | 49 (16.3) | 0.080 |
| Previous stroke | 27 (15.8) | 58 (19.3) | 0.34 |
| Atrial fibrillation | 42 (24.6) | 62 (20.6) | 0.32 |
| Current stroke event | | | |
| NIHSS score, median [IQR] | 16 [11 to 21] | 9 [6 to 15] | <0.001 |
| Prestroke mRS≥1, n (%) | 23 (13.5) | 47 (15.7) | 0.53 |
| Statin users, n (%) | 34 (20.9) | 96 (32.0) | 0.010 |
| Antithrombotic medications, n (%) |  |  |  |
| Aspirin | 52 (30.6) | 92 (30.6) | 1.00 |
| Clopidogrel | 7 (4.1) | 20 (6.6) | 0.26 |
| Anticoagulants | 23 (13.5) | 14 (4.7) | <0.001 |
| SBP, mean±SD, mmHg | 145±25 | 154±26 | <0.001 |
| DBP, mean±SD, mmHg | 79±14 | 82±15 | 0.021 |
| Biological data, median [IQR], | | | |
| LDL-C, g/L | 1.0 [0.8 to 1.3] | 1.1 [0.9 to 1.4] | 0.26 |
| Blood glucose, g/L | 1.2 [1.0 to 1.4] | 1.2 [1.0 to 1.5] | 0.71 |
| Wake-up stroke, n (%) | 29 (17.4) | 61 (20.3) | 0.45 |
| Any ICH, n (%) | 76 (44.2) | 52 (17.3) | <0.001 |
| HI 1 | 18 (10.5) | 12 (4.0) |  |
| HI 2 | 22 (12.8) | 22 (7.3) |  |
| PH 1 | 11 (6.4) | 4 (1.3) |  |
| PH 2 | 25 (14.5) | 14 (4.7) |  |
| DBP indicates diastolic blood pressure; HI, hemorrhagic infarction; ICH, intracranial hemorrhage; IQR, interquartile range; LDL-C, low-density lipoprotein cholesterol; mRS, modified Rankin scale; NIHSS, National Institutes of Health Stroke Scale; PH, parenchymal hematoma; SBP, systolic blood pressure; and SD, standard deviation. | | | |
